# Bat–man disease transmission: zoonotic pathogens from wildlife reservoirs to human populations

**DOI:** 10.1038/cddiscovery.2016.48

**Published:** 2016-06-27

**Authors:** N Allocati, A G Petrucci, P Di Giovanni, M Masulli, C Di Ilio, V De Laurenzi

**Affiliations:** 1 Department of Medical, Oral and Biotechnological Sciences, University ‘G. d'Annunzio’, Chieti, Italy; 2 Department of Pharmacy, University ‘G. d'Annunzio’, Chieti, Italy; 3 CESI-MET, University ‘G. d'Annunzio’, Chieti, Italy

## Abstract

Bats are natural reservoir hosts and sources of infection of several microorganisms, many of which cause severe human diseases. Because of contact between bats and other animals, including humans, the possibility exists for additional interspecies transmissions and resulting disease outbreaks. The purpose of this article is to supply an overview on the main pathogens isolated from bats that have the potential to cause disease in humans.

## Facts

- Bats are an important reservoirs of different pathogenic agents, and many of them have already caused disease outbreaks worldwide.
- More than 200 viruses have been associated with bats, and almost all are RNA viruses probably owing to their great ability to adapt to changing environmental conditions through a higher genetic variability.
- Bacteria in bats and their putative threat to humans remain poorly studied.

## Open questions

- The understanding of the mechanisms of viral persistence in bats remains unclear.
- Extensive studies are needed to improve our understanding of bat–human interactions in order to design new control measures in the future.
- Strategies on surveillance and monitoring of disease outbreaks in bat populations need to be further developed, in particular where bats and humans are in close contact.

Bats, mammals of the Chiroptera order, are present all over the world with the exception of the Arctic, the Antarctic and a few oceanic islands. Bats are the only mammals with the ability to fly and are present in >1100 different species.^1^ Bats are essential members of the global ecosystem and humans benefit from their presence in many ways. They are involved in seed dispersal and pollination activity: tropical bats are vital – as an example – in rebuilding cut down forests and in the pollination of wild plants as bananas, avocados and dates. Furthermore, the flying mammals are the major predators of night insects, including crop and human pests.^1^ Finally, their guano, which is rich in nitrogen, is used as biological fertilizer.

**Figure 1:**
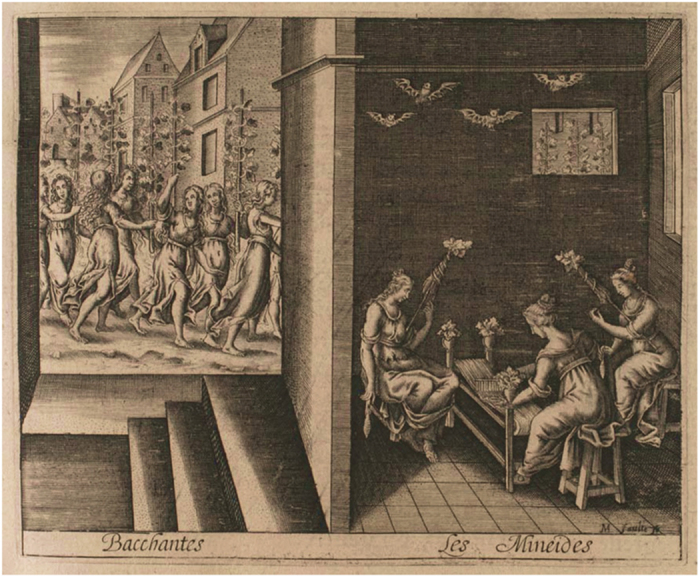
Bacchantes – Les Minéides. Illustration de Les Métamorphoses d’Ovide. Modified from Jean Mathieu, graveur; Ovide, auteur du texte. Editeur: veuve Langelier (Paris) – 1619. Source: gallica.bnf.fr – Bibliothèque nationale de France.

**Figure 2:**
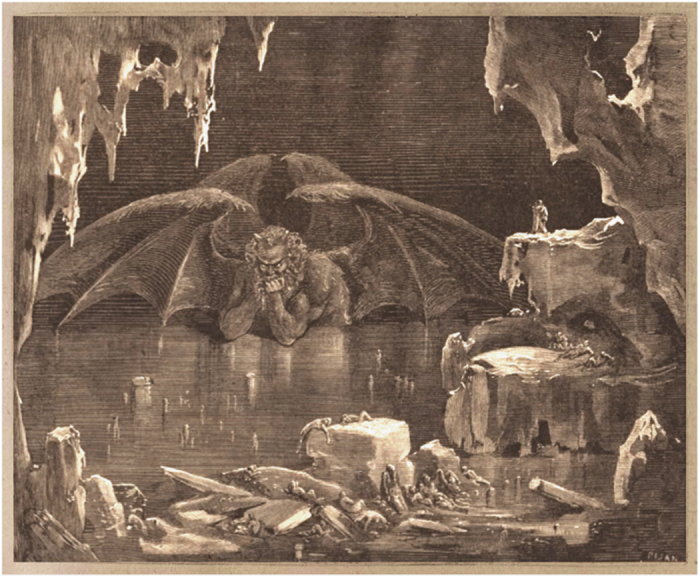
Satan. Modified from Dante Alighieri’s Inferno from the Original by Dante Alighieri and illustrated with the designs of Gustave Doré – 1861. Source: commons.wikimedia.org.

Despite the multiple benefits attributed to these animals, since the ancient times – through myths and misapprehensions – bats have gained a bad reputation in the general public. The classical literature is full of examples in which bats are associated with evil and darkness. The roman poet Ovid narrates in the Metamorphoses that the god Bacchus transforms the daughters of Mineus, king of Boeotia, into bats as a punishment for profanating his celebration (Figure 1 and Box A). In the Divine Comedy (Inferno – Canto XXXIV), Dante Alighieri, the father of the italian language, describes the devil Lucifer as bearing large bat wings (Figure 2 and Box B). In the eighteenth century, scientists called ‘vampire’ a bat that fed on blood, giving rise to the myth of human vampires that sucked blood from other men and could transform into bats. The Irish novelist Bram Stoker with his novel, Dracula (1897) did nothing but make this belief famous worldwide. More recently, Bob Kane, an American comic book artist, ideated a character called Batman a positive character that, however, disguises in a bat costume to scare his enemies: ‘*Criminals are a superstitious cowardly lot. So my disguise must be able to strike terror into their hearts. I must be a creature of the night, black, terrible..... ‘Just then a huge bat flies in the open window’. A bat! That’s it! It’s an omen. I shall become a bat! In: Detective Comics no 33, 1939.*

Bats, however, can be involuntarily dangerous to humans. Indeed, they are natural reservoir hosts and sources of infection for several microorganisms, including pathogens that can cause severe human diseases, and are more frequently implicated in zoonotic virus emergencies.^1,2^ Bats are widespread in urban areas and come in close contact with both domestic animals and humans, contaminating houses with guano and urine, additionally, humans occasionally encroach into bat habitats.^3^ Their characteristic ecology undoubtedly influences the maintenance and transmission of microorganisms within the colony and directly or indirectly to humans.^1,3^

Microbial transmission within bat colonies is promoted by the behavior of several species of these mammals aggregating in crowded roosts. Bats can transmit infectious agents to humans through intermediate hosts, which are in close contact with humans.^4^ These intermediate hosts can be infected in many ways, including ingestion of food partially digested by bats. Frugivorous bats, in fact, cannot ingest wide amounts of food because of the aerodynamics of flight,^5^ therefore, they extract nutrients by chewing fruits and spitting the residues. This partially digested food dropped on the ground can then be ingested by other animals and is a potential infectious source. A similar modality of viral transmission has also been described for insectivorous bats.^5^ Bats can also directly infect humans.^4^ This can occur through ingestion of infected bat meat, as in some areas bats are a food source, or through bat’s bite as in the case of rabies virus.

Other features of bat’s life make them a good host for infectious agents. The fact that several species of bats hibernate during the winter is one of these. Although the role of the hibernation in infection dynamics has not been widely studied, it is likely that this condition can contribute to the maintenance of pathogens in the cold weather (see below: white-nose syndrome (WNS)). Moreover, unlike many other small mammals, bats live >30 years. Their long life makes them a great reservoir for pathogens and gives them many occasions to transmit them to other species. In addition, some species of bats migrate – also for distances >1000 km – allowing them to spread diseases in big areas and acquire new microorganisms.^6,7^

Finally, most pathogens are not dangerous for bats and can therefore survive for long time in the host without killing it. Indeed, despite the fact that bats are infected with more different zoonotic viruses per host – with the exception of rabies virus and other *Lyssaviruses* – these are apparently not pathogenic for them, suggesting that they may control viral replication more efficiently than other mammals. It has been hypothesized that there may be a relationship between flight and low virulence.^8^ During flight, bats show an increase in the metabolic rate and in body temperature, comparable to the fever response rendering replication of infectious agents, which are temperature sensitive, less favorable.

In this review, we give a comprehensive overview of the microorganisms and viruses isolated from bats and their possible role in human disease.

## Viruses

Bats are recognized as important reservoirs of different families of viruses, most of which are emerging as human pathogens, such as Ebola and Marburg viruses, Severe Acute Respiratory Syndrome (SARS) and Middle East Respiratory Syndrome (MERS) coronaviruses. More than 200 viruses have been associated with bats, and almost all are RNA viruses probably owing to their great ability to adapt to changing environmental conditions through a higher genetic variability.^3,9^ In fact, RNA viruses have higher mutation rates compared with DNA viruses as the viral RNA polymerases lack proofreading activity. Furthermore, RNA viruses with segmented genomes have the ability to modify their genome through genetic re-assortment (i.e., *Orthomyxoviruses*). Below we report some examples of human infectious diseases associated with bat viruses.

### Rhabdoviridae

*Rhabdoviridae* contain six genera, including *Lyssavirus*, the most important bat-associated virus. At least 14 species of the *Lyssavirus* genus can be detected in bats, which are considered the ancestral hosts for these viruses. *Lyssaviruses* can be found worldwide and can be classified using different criteria, such as genetic distance, antigenic patterns, geographical distribution and host range.^10,11^ The characteristic bullet-shaped virus, transmitted to humans through the bite of infected animals, causes an acute, and frequently fatal, encephalitic disease.

The first report of a transmission of a viral disease from bats to humans was in 1911 and related to the rabies virus (RABV) belonging to the *Lyssavirus* genus.^12^ Carini^12^ suggested a link between rabies infection and hematophagous bats, known as vampires, in Central and South America. Several years later, rabies was also detected in non-hematophagous bat species.^13^ Although RABV is found worldwide in several terrestrial hosts, its presence in bats is observed only in the Americas. In Europe, four different *Lyssaviruses* have been isolated from bats: European Bat Lyssavirus type 1 (EBLV-1) and European Bat Lyssavirus type 2 (EBLV-2), Bokeloh Bat Lyssavirus (BBLV) and West Caucasian Bat Virus (WCBV).^14^ Recently, a new putative *Lyssavirus* in bat, named Lleida Bat Lyssavirus (LLBV), was found in Spain.^15^ To date, no human exposure to LLBV has been reported. EBLV-1, with the sub-types EBLV-1a and EBLV-1b, is the most isolated type throughout Europe. In addition, spillover infections by EBLV-1 in other mammals were also observed.^13,14^ The type 2 of EBLV is believed to be less virulent than type 1^13^ and is found less frequently being present only in few countries and human contamination has been reported only in two cases.^14^ Two other members of this family are found in bats but significantly less frequently than the previous ones: BBLV isolated in Germany and France^13,16,17^; WCBV isolated once in the Caucasus Mountains but also detected in Kenya in seropositive bats, suggesting a greater geographical distribution.^14,18^ Australian Bat Lyssavirus (ABLV) is the first endemic lyssavirus identified in Australia and is phylogenetically related to RABV and EBLV1.^10,19^ ABLV has been identified in all flying fox species on Australia’s land mass. Three fatal human infections by ABLV have been reported. Additionally, other viruses of this family detected in bats are summarized in Table 1.

### Paramyxoviridae

*Paramyxoviridae* constitute a wide viral family that includes human and animal pathogens. Several bat-borne paramyxoviruses have been recognized such as parainfluenza type 2 virus, Mapuera, Menangle and Tioman viruses and two infectious agents of emerging diseases, such as *Nipah* and *Hendra* viruses.^20^ *Nipah* and *Hendra* viruses, classified as the genus *Henipavirus*, are capable of causing severe, potentially fatal diseases in humans.^20^ Fruit bats of the *Pteropus* genus are the common reservoir hosts of the *Nipah* and *Hendra* viruses.^20^

*Nipah* virus (NiV) first emerged in 1998 in Malaysia, causing an outbreak of respiratory illness and encephalitis in pigs.^21^ Pig-to-human transmission of *Nipah* virus – associated with severe febrile encephalitis – was described and it was thought to occur through close contact with infected animals. Although uncommon, human-to-human transmission of virus was also described.^21^ In two other outbreaks in Bangladesh and India, an intermediate animal host was not identified, suggesting bat-to-human and human-to-human transmissions.

*Hendra* virus (HeV) causes a fatal respiratory disease in both humans and horses.^20,22^ Several outbreaks of HeV have occurred in Australia. Horse is the intermediate host and the virus is likely transmitted via ingestion of feed, pasture or water contaminated with urine, saliva and feces of infected bats. Horse-to-human transmission occurs when there is close contact with ill animals.^20^ To date, human-to-human transmission has not been observed.

### Coronaviridae

*Coronaviruses* (CoVs) prior to the SARS outbreak were only known to be the second cause of the common cold after rhinoviruses. At least four different species can cause mild, self-limiting upper respiratory tract infections in humans: alphacoronaviruses HCoV-229E and HCoV-NL63, and betacoronaviruses HCoV-HKU1 and HCoV-OC43. More recently, two more additional pathogenic human-CoV were identified: Severe Acute Respiratory Syndrome Coronavirus (SARS-CoV) and Middle East Respiratory Syndrome Coronavirus (MERS-CoV).^23^ SARS-CoV was first identified in China in February 2003, and 4 months later, >8000 cases had been reported with about 800 deaths in 27 different countries worldwide.^24^ SARS-CoV has a wide host range and it is associated with wildlife meat industry. The natural history of the virus involves bats as primary hosts that then transmitted it to the intermediate amplifying hosts – as mask palm civets and raccoon dogs – that then could spread it to humans.^23,25^ Human-to-human transmission follows and can lead to large numbers of infected patients and is considered the main route of transmission in large-scale epidemics.^9^

MERS-CoV is phylogenetically related to SARS-CoV and share with SARS-CoV the origin in bats.^23,26,27^ Several CoVs have been identified in insectivorous and frugivorous bat species in various countries, indicating that bats may represent an important reservoir of these viruses.^23^ MERS-CoV was first identified in Saudi Arabia in 2012 and then spread to other countries causing hundreds of deaths.^26,28^ Clinical features of MERS-CoV are similar to SARS-CoV, although this virus has also been associated with several extrapulmonary manifestations, such as severe renal complications. Recent studies have indicated that dromedary camels may be the intermediate hosts and potential source of the virus for humans.^26,29^ In addition, the first experimental infection of bats with MERS-CoV has been described. The virus maintains the ability to replicate in the host without clinical signs of disease, supporting the general hypothesis that bats are the ancestral reservoir for MERS-CoV.^30^ Human-to-human transmission has also been reported. Based on epidemiological data, both animal-to-human and human-to-human transmission are considered to be important elements in MERS outbreak.^26^

### Filoviridae

**Figure 3:**
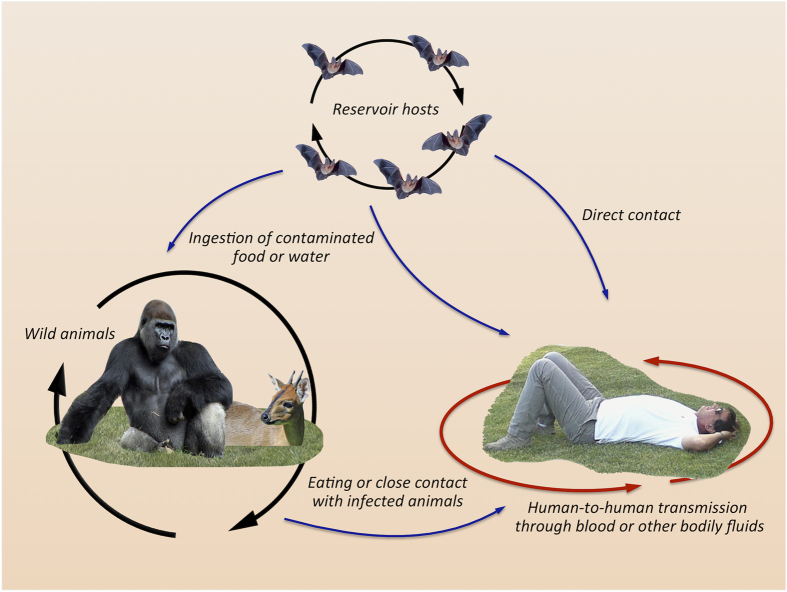
Schematic representation of Ebola virus transmission. Bats are the potential source of the virus. Infected bats can directly or through intermediate hosts spread the infection to humans. Human-to-human transmission can then result in epidemics.

*Ebolavirus* and *Marburgvirus* are two genera of the family *Filoviridae*, responsible for severe, often fatal, hemorrhagic fever diseases in humans and other primates.^31^ The first report on the *Marburgvirus* first was in 1967^32^ in German laboratory workers in Marburg who contacted it from African monkeys imported from Uganda. In 1976, a virus with similar characteristics but immunologically distinct was isolated in the Northern Democratic Republic of Congo and it was named *Ebolavirus*.^32^ Both viruses have generated several epidemics during the past years.^31^ Recently, in 2014, the largest ever registered Ebola epidemic started in West Africa and has affected several countries with >10 000 confirmed cases and thousands of deaths (Source CDC Atlanta, USA: 2014 Ebola outbreak in West Africa, updated 22 September 2015). The natural reservoirs for *Marburgvirus* and *Ebolavirus* are both fruit and insectivorous bat species, indicating that these filoviruses are multihost parasites.^31,33^ The virus is transmitted to humans through contact with body fluids – mainly blood and feces – and dead bodies of infected bats. Other animals such as monkeys and apes can also develop the disease and in turn transmit it to humans. Epidemics usually are a consequence of human-to-human transmission of the virus (Figure 3).

A third filovirus species of the new *Cuevavirus* genus, named *Lloviu* virus, was recently detected in insectivorous bats in Spain.^34^ *Lloviu* virus, genetically distinct from the others, is the first filovirus detected in Europe that was not imported from Africa. Unlike the other two species, this virus may be virulent in bats.^34^ As this virus has not been yet isolated, its capacity to infect other mammalian cells or to cause disease in humans remains to be determined.

### Orthomyxoviridae

*Orthomyxoviridae* are enveloped segmented RNA viruses that include five genera of which influenza A virus is the most preponderant pathogen in humans. It causes respiratory tract infections, resulting in moderate-to-severe disease and occasionally death. Influenza A viruses are divided into subtypes on the basis of two surface glycoproteins, namely, hemagglutinin (H) and neuraminidase (N). Influenza A virus is an uncommon promiscuous virus with a wide host range, including humans, pigs and birds. Recently, two new subtypes evolutionarily distinct from all others – H17N10 and H18N11 – were detected in different fruit bat species in Central and South America.^35,36^ It has been observed that, although H17N10 subtype is phylogenetically separate from all other subtypes, the virus genome is compatible with genetic exchange with human influenza A viruses, suggesting a potential re-assortment capability between subtypes and the consequent ability to generate highly pathogenic hybrid forms.^35^ More recently, serological evidence of influenza A virus subtypes other than H17N10 and H18N11 were reported in African frugivorous bats.^37^ In particular, about 30% antibody detection rate was found against avian H9 subtype known to cause infections in humans worldwide.^38^ These data, albeit preliminary, suggest that bats might represent asymptomatic mammalian carriers of influenza A viruses.^37^ Thus, similar to other pathogens, bats may represent a considerable reservoir for these viruses.

### Bunyaviridae

*Hantavirus* genus (from Hantan river in South Korea) is constituted of several emerging segmented RNA viruses that can cause human infections, including severe and lethal diseases such as hemorrhagic fever with renal syndrome and hantavirus cardiopulmonary syndrome.^39,40^ Rodents have long been believed to be the primary reservoirs of hantaviruses; however, a wider range of mammalian hosts, including insectivorous bats, has been reported.^39,40^ The evolutionary history of this genus is characterized by relatively frequent cross-species transmission that is also considered a major force in its evolution. The first hantavirus isolated from bats was the *Hantaan* virus, the etiological agent of hemorrhagic fever with renal syndrome.^41^ Successively, hantaviruses were identified in other bat species, but to date, however, no bat-to-human transmission of hantaviruses has been observed.^39^

### Reoviridae

*Mammalian orthoreovirus* of the genus *Orthoreovirus* can cause mild respiratory or gastrointestinal illness to severe diseases, including encephalitis and diarrhea. The virus is present in different serotypes throughout the world and has been isolated from several mammals, including humans.^42^ *Mammalian orthoreoviruses* were also isolated in several bat species, suggesting an extensive distribution of the virus in these animals.^42,43,44^ Several evidence suggest that bats may act as the natural reservoir of these viruses.^42^ Although bat-origin orthoreoviruses have been isolated from human patients, the zoonotic potential of these viruses is still unclear.^43,44,45^

### Other viruses

Several other mammalian viruses have been detected in bats for which the zoonotic potential or host range is unclear.^9,46–48^ An example are *Poxviruses* – important infectious agents of both humans and animals and capable of infecting multiple host species and to induce cross-species infections that were also recently identified in bats.^49^ Another example is the Dengue virus, an arthropod-borne virus belonging to the *Flavivirus* genus (*Flaviviridae*) that includes several relevant human pathogens associated with encephalitis and hemorrhagic fevers. Despite the fact that *Flaviviridae* are the second most frequent viruses found in bats and Dengue virus has been described in several bat species worldwide, the role of these animals in the dynamics of viral spreading remain insufficiently understood.^50^

## Bacteria

Unlike viruses, bacteria in bats and their putative threat to humans remain poorly studied.^51^ Here we report some examples of bacteria responsible for common human and animal infections that have occasionally been detected in bats.

### Bartonella spp.

Bartonellosis is a globally emerging zoonotic bacterial disease.^52^ *Bartonella* sp. is a Gram-negative bacterium transmitted through the bite of hematophagous arthropod vectors. Several species have been identified in domestic and wild animals, including bats.^53–55^ Recently, two species of *Bartonella* – *B. mayotimonensis* and *B. naantaliensis* – were detected from both the peripheral blood of bats and in their ectoparasites, suggesting that bats might be a source of the human bacterial pathogens.^54^ More recently, it has been reported the presence of closely related *Bartonella* genotypes in fruit bats and their associated bat flies in Madagascar, suggesting the transmission of a potentially zoonotic pathogen by bat fly vectors.^56^

### Pasteurella spp.

*Pasteurella* is commonly spread among animals as part of the normal microbiota of the oral, nasopharyngeal and upper respiratory tract.^57^ This genus comprises opportunistic pathogen species that can cause endemic disease and are associated with epizootic outbreaks.^57^ Animal bites and nasal secretions are the most likely sources of transmission to humans. In bats, various *Pasteurella* species – mainly *P. multocida* – have been identified as the main pathogens of several localized and systemic infections.^51,58^ The predominant source of infections appears to be wounds caused by the bite of domestic predators. However, a recent study from Wisconsin in USA reported for the first time an outbreak of acute pasteurellosis from *P. multocida* in wild bats without associated traumatic injures.^59^

### Leptospira sp.

*Leptospira* has worldwide distribution and its transmission to humans is primarily through exposure to water contaminated with the urine of infected animals.^60^ Bacterium harbors in several wild and domestic hosts, colonizes their kidneys and it is eliminated in their urine. The presence of *Leptospira* in bats has been demonstrated in several studies.^51,61,62^ However, the potential role of bats in human leptospirosis is questionable.^63,64^ In a case report, the patient’s history of bat exposure supports the idea that bats are a reservoir of the bacterium and can serve as a vector in disease transmission to humans.^64^

### Enterobacteriaceae

Several members of the *Enterobacteriaceae* family – responsible for a variety of human illnesses – were isolated from bats.^51,65–67^ A number of studies reported that *Salmonella* serotypes isolated from bats have similar characteristics to those found from livestock and humans, indicating that bats can be locally important in the epidemiology of salmonellosis in human and domestic livestock.^51,66^ Two of these serotypes, *S. typhimurium* and *S. enteritidis*, are a frequent cause of human and animal diseases.

*Escherichia coli* strain has also been frequently isolated from bats.^51,66–68^ It is to emphasize the high percentage of multiresistance of these class of pathogens to several classes of antimicrobials^51,66,68^ that is a major and increasing global health-care problem. Antimicrobial resistance was also observed in domestic and wild animals, with an increased incidence of resistance in both pathogenic and endogenous bacteria.^69^ Resistant pathogens can then be transmitted to humans and bats can therefore contribute to the spreading of resistant bacteria.

Several other genera – such as *Yersinia*, *Campylobacter*, *Vibrio* – have been identified in bats, but their impact on these animals remains mostly unknown.^51^

## Fungi

### Histoplasma capsulatum

*H. capsulatum* is a dimorphic pathogenic fungus of mammals, which causes pulmonary and systemic infections in humans and it is acquired via inhalation of the fungal spores. This microorganism is commonly found in soil associated with great amounts of birds’ droppings or bats guano. Although bats are considered the main reservoir and dispersers of this fungus in the environment, their role in spreading *H. capsulatum* remains unclear.^70^ It has, however, been observed that subjects occupationally exposed to bat sites, such as miners, geologists or farmers who use bat guano as fertilizer, have high risk of infection and can develop severe clinical forms of histoplasmosis.^70,71^

### Pseudogymnoascus destructans

Although implications in human health for this microorganism are not known, it is important to write a few words on an emerging fungal disease, named WNS, responsible for the deaths of millions of bats in North America. It is caused by the psychrophilic (cold-loving) fungus *P. destructans* that infects the skin of bats – especially the wings – during the winter months while they are in hibernation.^72^ Unlike other dermatophytes, which colonize the outer layer of the skin, *P. destructans* is able to invade the living tissue of the host causing the characteristic severe skin lesions.^73^ In addition *P. destructans* increases the frequency of periodic arousals in bats, resulting in premature consumption of stored fat essential to survive the winter leading to death within 4 months of infection.^72^ Recently, it has been observed that bacteria of the *Pseudomonas* genus – isolated from the skin of bats – inhibit the growth of the fungus *in vitro*.^74^ Additional *in vivo* studies will tell us whether in the future they could be used as biological control agents to protect bats exposed to *P. destructans*.

## Conclusions and Future Perspective

Emergence of new infectious diseases correlates with socio-economic, environmental and ecological factors and are a major public health problem as well as an important burden on economies worldwide.^75^ Most of these are caused by zoonotic pathogens originating in wildlife and then spreading to humans. Bats are an important reservoir of several pathogenic agents, mainly viruses, and many of them have already caused disease outbreaks worldwide. The increasing rate of bat-associated infections is also supported by an expanding overlap between bat and human habitats. Recently, to increase the knowledge of bat-associated viruses, a database has been constructed (http://www.mgc.ac.cn/DBatVir).^76^ DbatVir analyzes the virome diversity of bats as well as the ecological and epidemiological data to examine and track current and future bat-related transmissible diseases. To date, DbatVir has collected information on 5717 bat-associated animal viruses detected from 207 bat species in 77 different countries (update on 2 march 2016). Strategies on surveillance and monitoring of disease outbreaks in bat populations need to be further developed, in particular where bats and humans are in close contact. Extensive studies are also needed to improve our understanding of bat–human interactions to design new control measures in future. Furthermore, the identification of new human pathogens requires a continuous study to monitor the potential impact of these animals in their diffusion.

## Figures and Tables

**Table 1:** Overview of bat-associated infectious agents with zoonotic potential

*Pathogen*	*Diseases in human*	*Bat-to-human transmission has been observed*	*Reference*
*Viruses*			
Rhabdoviridae			
Rabies virus	Acute fatal encephalitis	Yes	Johnson *et al.*^13^
European Bat Lyssavirus type 1	Acute fatal encephalitis	Yes	McElhinney *et al.*^14^
European Bat Lyssavirus type 2	Acute fatal encephalitis	Yes	McElhinney *et al.*^14^
Bokeloh Bat Lyssavirus		No	Freuling *et al.*^16^
West Caucasian Bat virus		No	Kuzmin *et al.*^18^
Lleida Bat Lyssavirus		No	Aréchiga Ceballos *et al.*^15^
Australian Bat Lyssavirus	Acute fatal encephalitis	Yes	Weir *et al.*^19^
Aravan virus		No	Banyard *et al.*^10^
Khujand virus		No	Banyard *et al.*^10^
Irkut virus	Acute fatal encephalitis	Yes	Banyard *et al.*^10^
Lagos Bat Virus		No	Banyard *et al.*^10^
Duvenhage virus	Acute fatal encephalitis	Yes	Banyard *et al.*^10^
Shimoni bat virus		No	Banyard *et al.*^10^
Filoviridae			
*Ebola* virus	Ebola haemorrhagic fever	Yes	Olival and Hayman^31^
*Marburg* virus	Marburg haemorrhagic fever	Yes	Olival and Hayman^31^
Coronaviridae			
SARS-CoV	Severe Acute Respiratory Syndrome	Yes (palm civets, raccoon dogs)^a^	Drexler *et al.*^23^
MERS-CoV	Middle Eastern Respiratory Syndrome	Yes (camels)^a^	Drexler *et al.*^23^
Paramyxoviridae			
*Nipah* virus	Nipah disease (severe encephalitis)	Yes (pigs)^a^	Clayton *et al.*^20^
*Hendra* virus	Hendra disease (fatal respiratory disease)	Yes (horses)^a^	Clayton *et al.*^20^
Orthomyxoviridae			
*Influenza A* virus	Respiratory tract infections	No	Freidl *et al.*^37^
Bunyaviridae			
*Hantaan* virus	Fatal hemorrhagic fever	No	Holmes and Zhang^39^
Reoviridae			
Mammalian orthoreovirus	Enteric and respiratory infections	Unclear	Wang *et al.*^42^
			
*Bacteria*			
*Bartonella* spp.	Endocarditis	Unclear	Veikkolainen *et al.*^54^
*Pasteurella* spp.	Systemic infections	No	Mühldorfer^51^
*Leptospira* sp.	Systemic infections	Unclear	Vashi *et al.*^64^
*Salmonella* spp.	Salmonellosis	No	Mühldorfer^51^
*E. coli*	Several illnesses	No	Mühldorfer^51^
			
*Fungi*			
*H. capsulatum*	Pulmonary and systemic infections	Unclear	Santos *et al.*^71^

a Via intermediate host as indicated.

## Box A The daughters of Mineus are changed into bats

Iamque dies exactus erat, tempusque subibat, quod tu nec tenebras nec possis dicere lucem, sed cum luce tamen dubiae confinia noctis: tecta repente quati pinguesque ardere videntur lampades et rutilis conlucere ignibus aedes falsaque saevarum simulacra ululare ferarum, fumida iamdudum latitant per tecta sorores diversaeque locis ignes ac lumina vitant, dumque petunt tenebras, parvos membrana per artus porrigitur tenuique includit bracchia pinna; nec qua perdiderint veterem ratione figuram, scire sinunt tenebrae: non illas pluma levavit, sustinuere tamen se perlucentibus alis conataeque loqui minimam et pro corpore vocem emittunt peraguntque levi stridore querellas. tectaque, non silvas celebrant lucemque perosae nocte volant seroque tenent a vespere nomen. (Publius Ovidius Naso, Metamorphoses, book IV, lines 399–415)^1^.

^1^Now the day was past, and the time had come when you could not say that it was light or darkness, but a borderland of light and uncertain night. Suddenly the ceiling shook, the oil lamps seemed to brighten, and the house to shine with glowing fires, and fill with the howling of fierce creatures’ deceptive phantoms. Quickly the sisters hide in the smoke-filled house, and, in various places, shun the flames and light. While they seek the shadows, a thin membrane stretches over their slender limbs, and delicate wings enfold their arms. The darkness prevents them knowing how they have lost their former shape. They do not rise on soft plumage, but lift themselves on semi-transparent wings, and trying to speak emit the tiniest squeak, as befits their bodies, and tell their grief in faint shrieks. They frequent rafters, rather than woods, and, hating the light, they fly at night, and derive their name, ‘vespertiliones’, from ‘vesper’, the evening.

(Translated by AS Kline: www.poetryintranslation.com/).

## Box B Lucifer at the center of Cocytus

*Lo 'mperador del doloroso regno da mezzo 'l petto uscìa fuor de la ghiaccia*; *e più con un gigante io mi convegno, che i giganti non fan con le sue braccia:…….*

*……Sotto ciascuna uscivan due grand'ali, quanto si convenia a tanto uccello: vele di mar non vid'io mai cotali. Non avean penne, ma di vispistrello era lor modo; e quelle svolazzava, sì che tre venti si movean da ello:….*

(Dante Alighieri: La Divina Commedia, Inferno, Canto XXXIV)^2^.

^2^The emperor of the sorrowful kingdom stood, waist upwards, from the ice; and I am nearer to a giant in size than the giants are to one of his arms:…..

…..Under each face sprang two vast wings, of a size fit for such a bird: I never saw ship’s sails as wide. They had no feathers, but were like a bat’s in form and texture, and he was flapping them, so that three winds blew out away from him:…..

(Translated by AS Kline: www.poetryintranslation.com/).
